# The impact of effective dose to the immune cells on survival in breast cancer patients

**DOI:** 10.1016/j.ctro.2026.101251

**Published:** 2026-08-12

**Authors:** Stefanie A. de Boer, Daan S. Spoor, John H. Maduro, Pieter R.A.J. Deseyne, Christina T. Muijs, Nanna M. Sijtsema, Suzanne P.M. de Vette, Lisanne V. van Dijk, Ewoud Schuit, Johannes A. Langendijk, Anne P.G. Crijns

**Affiliations:** a University Medical Center Groningen, Radiation Oncology, Groningen, the Netherlands; b Julius Center for Health Sciences and Primary Care, University Medical Center Utrecht, Utrecht University, Utrecht, the Netherlands

**Keywords:** Breast cancer-specific survival (BCSS), Effective dose to immune cells (EDIC), Adjuvant radiation therapy

## Abstract

**Purpose:**

In patients with thoracic cancers such as lung and esophageal cancer, higher radiation exposure to immune cells is associated with worse survival outcomes. This study aims to determine whether a similar association between Effective Dose to the Immune Cells (EDIC) and BC-specific survival (BCSS) also exists in BC patients.

**Methods:**

This retrospective cohort study included stage I to III invasive BC patients treated with adjuvant radiation therapy (RT) between January 2005 and June 2015. The EDIC was calculated as the equivalent uniform dose of the mean heart dose, mean lung dose and large and small blood vessels. The association between EDIC and BCSS was assessed using univariable and multivariable Cox proportional hazard regression models.

**Results:**

The study included 3423 BC patients (median age of 59 years, IQR 50–67) with a median follow-up of 7.7 years (IQR, 5.5–10.8). Overall, 710 patients (20.7%) died, including 323 (9.4%) from BC. The median EDIC was 2.1 Gy (IQR, 1.6–2.7). Higher EDIC, particularly >3.0 Gy, was associated with worse BCSS. In multivariable analysis, EDIC was independently associated with BC mortality (HR 1.66; 95%CI 1.44–1.91), consistent with worse BCSS.

**Conclusions:**

An association between higher EDIC and worse BCSS was observed in patients treated with adjuvant RT, with the association being most pronounced at EDIC levels >3.0 Gy. We hypothesize that this effect may be mediated by radiation-induced lymphopenia; however, whether strategies aimed at reducing EDIC translate into improved oncological outcomes requires prospective validation.

## Highlights

• This study included 3423 stage I–III breast cancer patients treated with adjuvant radiotherapy.
• Higher Effective Dose to the Immune Cells (EDIC) was independently associated with worse breast cancer–specific survival.
• Most patients (>80%) had EDIC <3 Gy, the highest risk of worse BCSS observed in those with EDIC >3 Gy.
• EDIC may represent a prognostic marker in breast cancer radiotherapy.

## 1 Introduction

Most women diagnosed with breast cancer (BC) have a favourable prognosis. Adjuvant radiotherapy (RT) is a cornerstone of treatment, reducing the risk of recurrence after surgery [1], [2], but it may also cause radiation-induced complications. The risk of radiation-induced cardiovascular disease (CVD) increases with higher mean heart dose (MHD) [3], [4], [5]. Studies on thoracic cancers, including lung and esophageal cancer, consistently show that higher heart and lung dose exposure is associated with worse overall survival (OS) [6], [7], [8].

More recently, the immune system has been recognized as an organ at risk (OAR) [9], [10]. Radiation-induced lymphopenia (RIL) is one of the most common immunosuppressive effects of RT as lymphocytes are highly radiosensitive [11]. Several studies suggest that RIL is associated with higher incidence of locoregional recurrence (LRR), worse overall survival (OS) and reduced progression-free survival (PFS) [9], [12], [13], [14], [15]. In addition, among patients with lung and esophageal cancer, higher Effective Dose to the Immune Cells (EDIC) correlates with worse PFS and OS [16], [17], [18]. However, the impact of EDIC on survival outcomes in BC remains to be determined [19]. Therefore, the primary aim of this study was to investigate if EDIC is independently associated with breast cancer-specific survival (BCSS) in a large cohort of BC patients treated with adjuvant RT. Secondary aims were to evaluate the impact of EDIC on OS, LRR, and distant metastasis (DM).

## 2 Methods

### 2.1 Study population

All women aged ≥18 years with stage I–III invasive BC who started adjuvant RT between January 2005 and June 2015 at the University Medical Center Groningen (UMCG) and had available CT-based planning data were eligible. Exclusion criteria were bilateral, metastatic or recurrent disease at diagnosis, prior cancer (except for nonmelanoma skin cancers), previous or partial RT, partial breast RT and treatment within a research setting applying an atypically high boost dose, which was excluded from the analyses.

### 2.2 Data collection

Medical records were retrospectively reviewed to retrieve information on patient, tumour, and treatment characteristics, follow up data as well as information on comorbidities and cardiovascular (CV) risk factors. If patients received neoadjuvant therapy, clinical T- and N-stage data were used: otherwise pathological T- and N-stage data were utilized. To compensate for missing data and to obtain the most recent follow-up data, additional questionnaires were sent to general practitioners (GPs) [5]. This procedure was approved by the medical ethics committee of the UMCG (METc 2017.231).

### 2.3 Data definitions

Follow-up extended from the first day of RT until any event or last reliable follow-up (medical visits or GP records). Survival status was verified using the national Personal Records Database (PRD). BC-specific death was defined as death from active or progressive disease, determined by medical record review; unclear cases were independently reviewed by two physicians (SdB/AC). LRR was recurrence disease in the ipsilateral breast, chest wall, or regional lymph nodes; DM was any distant metastasis.

### 2.4 Radiation dosismetry and treatment

3D conformal RT using CT-based planning was delivered, as described previously [3], [20]. Various RT schedules were used over the ten-year inclusion period (Table 1). Locoregional lymph nodes irradiation depended on axillary dissection and pathologic risk factors, according to evolving protocols.

**Table 1:** Patient clinical characteristics at baseline (*n* = 3423) by BCSS.

Characteristic	Overall population (N = 3423)	Died from BC (N = 323)	Did not die from BC (N = 3100)	*P-value*
Age at BC diagnosis, years				
Median, IQR	59, 50–67	56 (46–67)	59 (50–67)	0.029
Categorized, n (%)				
18–35	75 (2.2)	20 (6.2)	55 (1.8)	< 0.001
36–45	394 (11.5)	56 (17.3)	338 (10.9)	
46–55	955 (27.9)	82 (25.4)	873 (28.2)	
56–65	991 (29.0)	79 (24.5)	912 (29.4)	
65–75	824 (24.1)	60 (18.6)	764 (24.6)	
> 75	184 (5.4)	26 (8.0)	158 (5.1)	

T stage, n (%)				
T1	2267 (66.2)	124 (38.4)	2143 (69.1)	< 0.001
T2	926 (27.1)	130 (40.2)	796 (25.7)	
T3	143 (4.2)	37 (11.5)	106 (3.4)	
T4	87 (2.5)	32 (9.9)	55 (1.8)	

N stage, n (%)				
N0i+	2133 (62.3)	117 (36.2)	2016 (65.0)	< 0.001
N1	837 (24.5)	88 (27.2)	749 (24.2)	
N2	283 (8.3)	59 (18.3)	224 (7.2)	
N3	170 (5.0)	59 (18.3)	111 (3.6)	

Laterality of the breast, n (%)				
Right	1641 (52.1)	145 (44.9)	1496 (48.3)	0.266
Left	1782 (47.9)	178 (55.1)	1604 (51.7)	

Tumour grade, n (%)				
Grade 1	806 (23.6)	26 (8.1)	780 (25.1)	< 0.001
Grade 2	1562 (45.6)	117 (36.3)	1445 (46.6)	
Grade 3	1055 (30.8)	180 (55.6)	875 (28.2)	

Lympho-vascular space invasion, n (%)				
Yes	637 (18.6)	128 (39.7)	508 (16.4)	< 0.001
No	2786 (81.4)	195 (60.3)	2592 (83.6)	

Subtype, n (%)				
HR positive / HER2 negative	2565 (74.9)	190 (58.9)	2374 (76.6)	< 0.001
HER2 positive	454 (13.3)	51 (15.8)	403 (13.0)	
Triple negative	404 (11.8)	82 (25.3)	323 (10.4)	

Chemotherapy, n (%)				
Yes	1648 (48.1)	225 (69.7)	1423 (45.9)	< 0.001
No	1775 (51.9)	98 (30.3)	1677 (54.1)	

Current smoker or ex-smoker, n (%)				
Yes	1947 (56.9)	186 (57.5)	1762 (56.8)	0.490
No	1476 (43.1)	137 (42.5)	1338 (43.2)	

Presence of 1 or more cardiovascular risk factors (defined as diabetes, obese (BMI > 30), hypertension or hypercholesterolemia, n (%)				
Yes	1622 (47.4))	154 (47.8)	1468 (47.4)	0.670
No	1801 (52.6)	169 (52.2)	1632 (52.6)	

Previous cardiovascular disease, n (%)				
Yes	513 (15.0)	47 (14.6)	466 (15.0)	0.468
No	2910 (85.0)	276 (85.4)	2634 (85.0)	

Radiation treatment, n (%)				
16 × 2.66 Gy	604 (17.6)	19 (5.9)	585 (18.9)	< 0.001
21 × 2.17 (SIB 2.66) Gy / 23 × 2.03 (SIB 2.66) Gy	697 (20.4)	34 (10.5)	663 (21.4)	
25 × 2 Gy	502 (14.7)	124 (38.4)	378 (12.2)	
28 × 1.8 Gy (SIB 2.3 or 2.4 Gy)	1620 (47.3)	146 (45.2)	1474 (47.5)	

Abbreviations: IQR, interquartile range; BMI, body mass index; SIB, Simulated Integrated Boost; BC, Breast cancer.

Dose-volume parameters for the heart, the lungs and external contour were obtained from nominal 3D dose distributions after contouring with an in-house-developed multi-atlas-based automated segmentation (Mirada Workflow Box v2.0) [21]. Integral total dose volume (ITDV) was calculated as the mean external dose (MED) multiplied with external contour volume, with CT scans extending from skull base to first lumbar vertebra, including all parts of the patient that received RT.

### 2.5 Effective dose to immune cells (EDIC)

EDIC was estimated using the Jin et al. model, previously described in detail [18], [22], which approximates that RT dose to circulating blood serves as a proxy for EDIC, assuming continuous blood flow through all organs [23]. BX% represents the fraction of total blood volume in each organ. For organs with high blood volume (lungs, heart, large blood vessels), the equivalent uniform dose (EUD) is approximately equal to mean organ dose (MOD)*B%; i.e. for lungs B1% ≈ 0.12, for heart B2% ≈ 0.08, and for large blood vessels B3% ≈ 0.45. For the small blood vessels B3% ≈ 0.35; since they have a slower blood flow a dose effectiveness factor (k1 = 0.85) is included and fraction number (n) is included: the EUD to blood in the small blood vessels is then estimated by ∼ MOD*B%*k1*($\frac{n}{45}$)^1/2^. The EDIC is the sum of the contributions from all major blood-containing organs within the thorax based on anatomy as previously described [18]. The average dose to large and small vessels—organs not routinely contoured as OARs—was estimated using the integral dose (ID), assuming uniform distribution. EDIC was calculated as follows:

$$\mathrm{EDIC}=B1\%\times \mathrm{MLD}+B2\%\times \mathrm{MHD}+\left[B3\%+B4\%\times k1\times {\left(\frac{n}{45\;}\right)}^{\frac{1}{2}}\right]\times \frac{\mathrm{ITDV}}{61.8\times 10^{3}}$$

The ITDV was calculated as described above and corrected for mean body volume of 61.8 × 10^3^ cm^3^, assuming an average body weight of 63 kg, to calculate the ID in line with previous studies [10], [18].

### 2.6 Study endpoints

The primary endpoint was BCSS. Secondary included OS, LRR, and DM. In addition, as the EDIC is the sum of the contributions from MHD, mean lung dose (MLD) and ID, the effects of dose on these OARs on the primary and secondary endpoints were also examined separately. EDIC effects on BCSS were illustrated using quartiles and equal 1.5 Gy dose increments. Furthermore, the dosimetric impact of BC laterality was also examined as a correlation between OAR dose and BC laterality. Baseline patient and tumour characteristics were compared across EDIC categories.

### 2.7 Statistical analysis

We used multiple imputation by chained equations (MICE) (10 times) to account for the missing clinical data. The imputation model included all covariates, confounders, and outcomes studied. All analyses described above were performed in each imputation set separately and afterwards combined using Rubin's rules [24]. Dose-volume parameters had <1% missing and were not imputed.

Univariable and multivariable Cox proportional hazard regression models evaluated associations between RT parameters (EDIC, MHD, MLD, ID) and outcomes (BCSS, OS, LRR, DM). The BCSS event was death due to BC. The functional forms of the association between different continuous treatment factors and endpoints were explored by multifractional polynomials and restricted cubic spline plots to account for potential non-linear associations: only MLD for OS required a squared transformation.

In the multivariable Cox regression models, we adjusted for potential confounders related to BC recurrence and premature death, including age, tumour characteristics (T and N classification, laterality, tumour grade, molecular subtype, lymphovascular invasion), chemotherapy, and RT schedule. Because BC and CVD share risk factors we also adjusted for smoking (yes/no), presence of 1 or more cardiovascular risk factors (including diabetes, obesity (BMI > 30), hypertension or hypercholesterolemia), and previous CVD [25], [26], [27]. Group differences were tested with Chi-Square or Mann-Whitney *U* tests, survival with Kaplan-Meier and log-rank, and correlations with Spearman's coefficient. Cox analyses were performed in R (v4.1.2); other analyses in SPSS (v28).

## 3 Results

### 3.1 Patient characteristics

Of the 5752 patients treated with RT, 3423 met the eligibility criteria (Supplemental Fig. 1). Most exclusions were due to lack of informed consent for participation in the institutional research cohort (*N* = 702), incomplete planning-CT data (*N* = 564) or non-invasive BC (*N* = 397). Median age was 59 years; most had T1 (66.2%) and N0 (62.3%). About half the patients received chemotherapy (48.1%) and/or hormone therapy (56.8%), 116 (3.4%) patients received neoadjuvant therapy, BC laterality was evenly distributed. Patients' characteristics are summarized in Table 1.

### 3.2 Endpoints

Median follow-up was 7.7 years (IQR, 5.5–10.8 years). Out of the 3423 patients in the cohort, 710 died (20.7%), 323 of which due to BC (9.4%). The 5-year and 10-year cumulative BCSS survival rates were 93.5% and 86.6%, respectively. The 5-year and 10-year cumulative OS rates were 89.3% and 77.3%, respectively. BC specific mortality occurred at a median time of 4.2 years after RT (IQR, 2.2–6.2), overall mortality occurred at a median time of 4.8 years after RT (IQR, 2.8–7.7), LRR occurred in 134 (3.9%) patients and 396 (11.6%) patients developed DM.

### 3.3 Effect of clinical characteristics on BCSS

Differences in clinical characteristics between patients that died from BC (*n* = 323) or not (*n* = 3100) are shown in Table 1. As expected, tumour characteristics (T classification, N classification, tumour grade, molecular subtype (ER/PR+ and HER2 negative, HER2 positive, and triple negative) and lymph vascular invasion (yes/no) were significantly different between the two groups. Also, RT schedule, age, and chemotherapy (yes/no) were significantly different. BC laterality (right-sided vs left-sided) was not significantly different between patients who died from BC and those who did not. Common risk factors for CVD and cancer like smoking status, presence of 1 or more CV risk factor and previous CVD were not significantly different between patients who died from BC and those who did not.

### 3.4 Radiation dose OARs

For the total population the median EDIC was 2.08 Gy (IQR, 1.64–2.72 Gy), the median MHD was 1.80 Gy (IQR, 1.18–3.60 Gy), the median MLD was 4.70 Gy (IQR, 3.56–6.40 Gy) and the median ID was 1.95 Gy (IQR, 1.44–2.60 Gy). The RT dose on OARs based on laterality of the breast was not significantly different for EDIC, but was significantly different for MHD, MLD and ID, see Table 2. As expected, EDIC was found to correlate with MHD, MLD, and ID with the strength of the correlation increasing when the population was stratified by BC laterality (Supplementary Table 1).

**Table 2:** OAR dose based on laterality of the BC

Variable	Total population		Left-sided BC		Right-sided BC		*P*
	Total cases	Median (IQR)	Total cases	median, (IQR)	Total cases	Median, (IQR)	
MHD (Gy)	3423	1.80 (1.18–3.60)	1782	3.38 (2.09–5.11)	1641	1.20 (0.95–1.53)	< 0.001
MLD (Gy)	3403	4.70 (3.56–6.40)	1772	4.05(3.16–5.50)	1631	5.51 (4.33–7.25)	< 0.001
ID (Gy)	3410	1.95 (1.44–2.60)	1774	1.89 (1.42–2.56)	1636	2.02 (1.49–2.66)	0.001
EDIC (Gy)	3391	2.08 (1.64–2.72)	1764	2.06 (1.61–2.70)	1627	2.11 (1.66–2.75)	0.201

Abbreviations: IQR, interquartile range; MHD, mean heart dose; MLD, mean lung dose; ID, integral dose; EDIC, Effective Dose to Immune Cells.

### 3.5 Effect of EDIC on BCSS

**Fig. 1:**
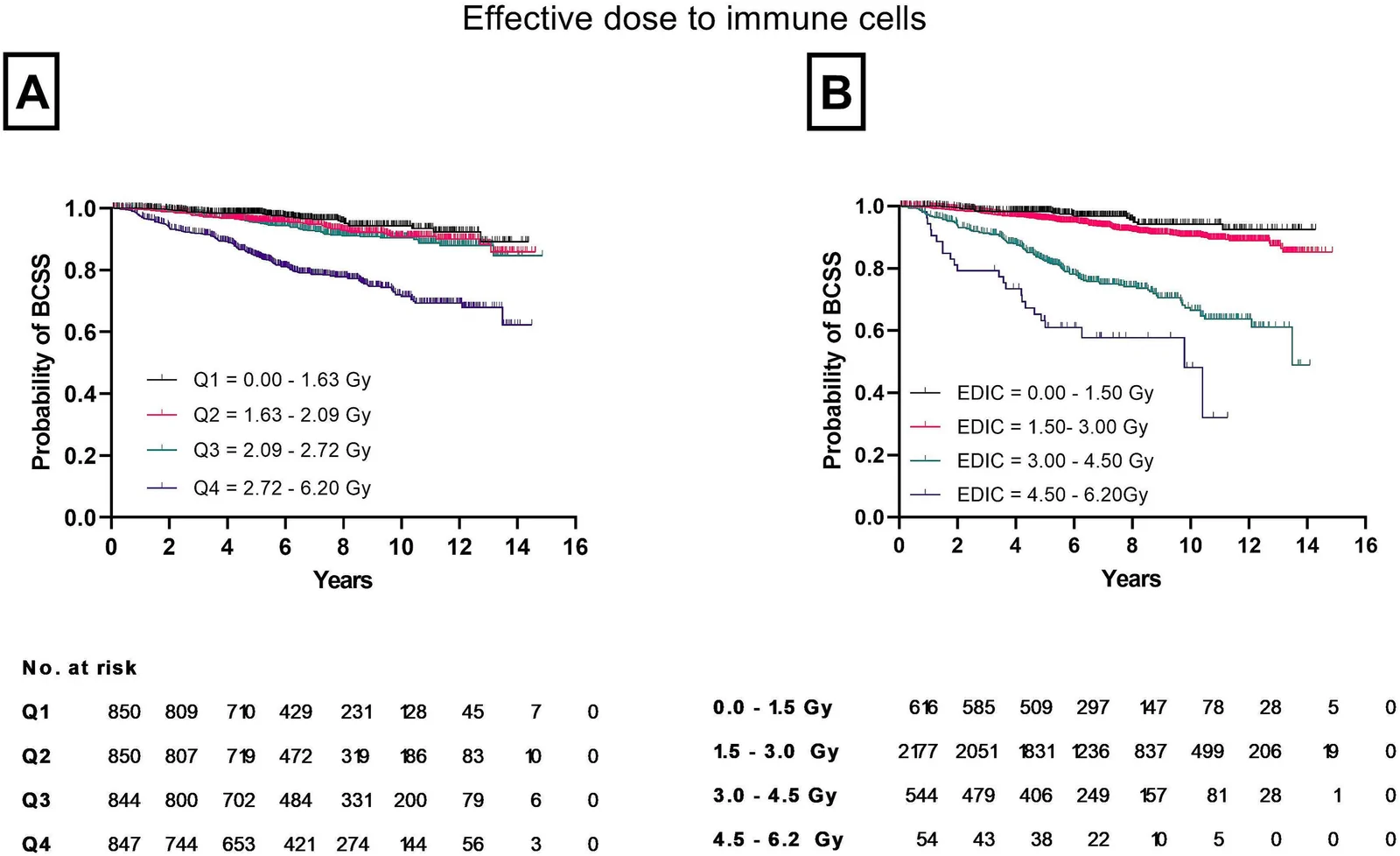
Effective dose to immune cells. Kaplan–Meier curves for breast cancer–specific survival (BCSS) comparing patients by EDIC quartiles (A) and by absolute EDIC values grouped in equal increments of 1.5 Gy (B).

Kaplan-Meier curves showed that BCSS decreased significantly by increasing quartiles of EDIC (Fig. 1A, log-rank *P* < 0.001). For patients in Q1 (i.e., EDIC: 0.00–1.63 Gy), the BCSS was 98.4% at 5 years and 94.3% at 10 years. In contrast, for patients in Q4 (i.e., EDIC: 2.72–6.20 Gy), the BCSS was 84.6% at 5 years and 71.5% at 10 years. Kaplan-Meier curves showed a significant decline in BCSS with increasing EDIC, as observed in patient groups categorized by equal EDIC increments of 1.5 Gy (Fig. 1B, log-rank *P* < 0.001). This effect was most pronounced for patients with an EDIC >3.0 Gy. For patients with an EDIC of 3.0–4.5 Gy, the BCSS was 82.2% and 66.5% at 5 and 10-years, respectively. For patients with an EDIC of 4.5–6.2 Gy, the BCSS was even lower; 63.2% and 48.1% at 5 and 10 years. However, most patients (*N* = 2793, 82%) had an EDIC of less than 3 Gy.

Baseline patient and tumour characteristics differed across EDIC categories (Supplementary Table 2). Higher EDIC was associated with higher T stage, higher N stage, higher tumour grade, presence of lymphovascular invasion, and more frequent administration of chemotherapy (all *p* < 0.001). Age distribution also differed between groups (p < 0.001), whereas laterality did not (*p* = 0.181).

In Cox regression, with death by BC as the event, higher EDIC was associated with increased BC mortality both univariably (HR 2.26, 95% CI 2.04–2.51, p < 0.001) and after multivariable adjustment (HR 1.66, 95% CI 1.44–1.91, p < 0.001), indicating worse BCSS with rising EDIC (Table 3).

**Table 3:** Univariable and multivariable Cox regression models for different OARs and endpoints.

					Univariable model		Multivariable model⁎	
Organ at risk	Variable	End-point	Total cases	No. Events	HR (95% CI)	p-value	HR(95% CI)	p-value

Immune Cells	EDIC, continuous	BCSS	3391	320	2.26 (2.04–2.51)	<0.001	1.66 (1.44–1.91)	<0.001
		OS	3391	701	1.72 (1.59–1.86)	<0.001	1.32 (1.19–1.47)	<0.001
		LRR	3391	132	1.64 (1.37–1.97)	<0.001	1.68 (1.32–2.13)	<0.001
		DM	3391	392	2.07 (1.88–2.28)	<0.001	1.61 (1.41–1.84)	<0.001

Heart	MHD, continuous	BCSS	3423	323	1.16 (1.12–1.21)	<0.001	1.14 (1.08–1.21)	<0.001
		OS	3423	710	1.08 (1.05–1.12)	<0.001	1.07 (1.03–1.12)	<0.001
		LRR	3423	134	1.12 (1.06–1.20)	<0.001	1.17 (1.08–1.28)	<0.001
		DM	3423	396	1.13 (1.08–1.17)	<0.001	1.11 (1.05–1.17)	<0.001

Lung	MLD, continuous	BCSS	3403	320	1.30 (1.25–1.35)	<0.001	1.16 (1.09–1.23)	<0.001
		OS⁎⁎	3403	703	3.08 (2.55–3.72)	<0.001	1.76 (1.32–2.35)	<0.001
		LRR	3403	133	1.21 (1.14–1.30)	<0.001	1.29 (1.18–1.41)	<0.001
		DM	3403	392	1.28 (1.24–1.33)	<0.001	1.17 (1.11–1.23)	<0.001

Body	ID, continuous	BCSS	3410	322	1.92 (1.75–2.12)	<0.001	1.47 (1.30–1.67)	<0.001
		OS	3410	707	1.59 (1.48–1.70)	<0.001	1.23 (1.12–1.35)	<0.001
		LRR	3410	133	1.39 (1.17–1.66)	<0.001	1.32 (1.07–1.63)	<0.001
		DM	3410	395	1.79 (1.63–1.95)	<0.001	1.43 (1.27–1.60)	0.01

Abbreviations: EDIC, Effective Dose to Immune Cells; MHD, mean heart dose; MLD, mean lung dose; ID, integral dose; LRR, locoregional recurrence; DM, distant metastasis; BCSS, breast cancer-specific survival; OS, overall survival. HR, Hazard Ratio; CI, Confidence Interval.

⁎ Adjusted for: T classification, N classification, BC laterality, tumour grade, molecular subtype (ER/PR+ and HER2 negative, HER2 positive, and triple negative), lymph vascular invasion (yes/no), RT schedule, age in categories, chemotherapy (yes/no), current smoking (yes/no), presence of 1 or more cardiovascular risk factors (defined as diabetes, obese (BMI > 30), hypertension or hypercholesterolemia), and previous cardiovascular disease (yes/ no).

⁎⁎ Data transformed ((MLD/10^2).

### 3.6 Secondary endpoints

Besides EDIC also MHD, MLD and ID were associated with worse BCSS, and RT dose to all OARs (EDIC, MHD, MLD, ID) predicted worse OS, higher LRR, and increased DM in both univariable and multivariable analyses (Table 3). Kaplan-Meier curves showed that BCSS decreased significantly by increasing quartiles of MHD, MLD and ID (Supplemental Fig. 2, all log-rank *P* < 0.001).

## 4 Discussion

This large retrospective cohort study showed a significant association between EDIC and worse BCSS even after adjustment for the most relevant confounding factors like tumour characteristics (T and N classification, tumour grade, etc.), age and shared common risk factors for CVD and cancer, this association remained significant with a 66% higher risk of dying from BC with each increase of 1 Gy EDIC. Furthermore, MHD, MLD and ID are also associated with worse BCSS. These results are in line with observations among other thoracic tumour sites suggesting that irradiation of heart, lungs and immune cells negatively affect OS and cancer-specific survival. [6], [7], [8], [10], [16], [17], [28]

Interestingly, the current study showed that BC laterality is not different between patients who died from BC and those who did not, while the dose to OARs, like heart and lungs, is significantly different. As expected, due to the anatomical configuration, patients with left-sided BC have a higher MHD and lower MLD compared to right-sided BC patients [29], [30]. Because EDIC is a composite metric that integrates multiple DVH parameters, opposing laterality-associated differences in MHD and MLD may partially offset each other, resulting in comparable EDIC values across BC laterality. Accordingly, while individual organ-at-risk dose metrics remain clinically relevant and routinely reported, EDIC may be the optimal parameter for evaluating the association between RT to IC and oncologic outcome. In support of this concept, a recent study by Yu *et al* [31] showed that ID was the most critical factor contributing to RIL among all investigated dose-volume parameters, including MLD and MHD. This finding suggests that larger irradiated volumes, in themselves, are a risk factor for worse oncologic outcomes. Indeed, growing evidence indicates that (low dose) RT may have systemic pro-oncogenic effects [32], [33]. One potential mechanism underlying these effects is RIL, as RIL is associated with worse tumour response, PFS and OS [6], [7], [8], [16], [17], [18]. Also in BC treatment, in patients with triple-negative BC, systemic inflammation and impaired immune status following RT were found to be independent predictors of LRF, PFS and OS. Higher post-RT absolute lymphocyte count was associated with reduced LRR and improved PFS [14]. Furthermore, a relative decline in peripheral lymphocyte count (nadir pre- < 0.8) correlated with worse DFS. In a post-hoc analysis of a randomized controlled trial, hypofractionated RT (43.5 Gy/15 fractions) resulted in a lower risk of lymphopenia, compared to conventional RT (50 Gy /25 fractions) [19].

Accurately estimating RT dose to the immune system is challenging because circulating lymphocytes are constantly moving. To address this, a model estimating EDIC in circulating blood during thoracic RT was developed in NSCLC [18], [22] and later validated in NSCLC [34] and esophageal cancer [35], [36]. More recently, it was applied in BC patients, showing a significant correlation between EDIC and RIL [10]. In that study, most patients received hypofractionated RT (15 fractions). Although the EDIC model was originally designed for >20 fractions [18], [22], the study suggested potential applicability in hypofractionated BC patients [10]. Interestingly, the same study also showed that the corresponding EDIC to induce 50% of grade-1, grade-2 and grade-3 RIL was 1.2, 2.1 and 3.7 Gy [10]. In the current study, the effect on BCSS was most pronounced in patients with an EDIC greater than 3 Gy. The observed HR indicates that each 1 Gy increase in RT dose is associated with a higher risk of BC-related death, suggesting an overall linear dose–response relationship. Spline analyses supported this trend, with deviations at the lower and higher dose ranges. Kaplan–Meier curves showed overlapping survival for EDIC values between 0 and 3 Gy, with minimal mortality, suggesting limited RT impact on BCSS within this range. However, above 3 Gy, the curves diverged, indicating a possible threshold for increased risk, possibly due to higher rate of severe RIL. This is in line with several other studies in patients with thoracic cancer in which severe lymphopenia, grade III or higher, was a predictor for poor PFS and OS [9], [12], [13], [37], [38], [39].

Compared to lung and esophageal cancer, RT dose distributions to OARs in BC patients are different. Currently, BC planning primarily focuses on minimizing heart dose to reduce radiation-induced CVD [3], [4]. Techniques such as deep-inspiration breath hold, IMRT/VMAT have already reduced the MHD [40], [41] and proton RT can further lower dose to the heart, lungs and subsequently ID and EDIC [39], [42], [43], [44], [45]. In addition, hypofractionated RT with a reduction in circulating blood dose also appears to decrease the risk of RIL [19]. Strategies to limit immune cell dose might further improve outcomes, but causality cannot be inferred, and reducing EDIC may not directly translate into survival benefit. With the increasing use of immunotherapy in BC patients, such as pembrolizumab combined with chemotherapy for triple-negative BC, which has shown improved PFS [46], minimizing EDIC could be particularly relevant given its potential impact on the immune system. The prognostic impact of EDIC may differ across molecular subtypes because of differences in tumour immunogenicity. While molecular subtype was adjusted for in the multivariable analyses, subtype-specific effects remain to be investigated. However, in the current cohort, more than 80% of patients had an EDIC <3 Gy. Therefore, most BC patients already receive relatively low OAR doses, and only a specific subset of patients may benefit from these advanced approaches.

In contrast to lung and esophageal cancer, where the tumour remains in situ and GTV/PTV also reflects tumour burden, our models incorporated detailed tumour characteristics such as T and N stage, grade, molecular subtype, and lymphovascular invasion as oncologic risk factors. Notably, higher EDIC levels were observed more frequently in patients with adverse tumour characteristics; however, these factors were explicitly accounted for in the multivariable analyses. In addition, we adjusted for cardiovascular and cancer-related risk factors (e.g., smoking, obesity, hypertension, diabetes, hypercholesterolemia, and prior CVD), which represent important competing risks for non–cancer death [25], [26], [27]. Together, these adjustments strengthen the completeness of our models.

An important limitation of the current study is that we did not measure peripheral lymphocyte count, so we could not evaluate the direct impact of lymphopenia on BCSS and OS. Instead, we evaluated its association using EDIC as a surrogate dosimetric measure [10]. Therefore, the biological mechanism linking EDIC to clinical outcome could not be directly evaluated in this cohort. However, the EDIC model estimates the dose to the circulating immune cells based on assumptions such as using ID to calculate the dose to the vessels and assuming that the percentage of blood volumes accurately reflect the blood distribution in the body [18], [22]. Nevertheless, the lung and heart volumes are accurate and are the most important contributors of the model. Moreover, the mean liver dose was not included in the EDIC model because the CT scans did not cover the whole liver and there is no consensus on its inclusion [10], [18], [35], [47]. Furthermore, baseline immune-related characteristics, such as autoimmune disease, chronic infections, corticosteroid use, and other laboratory markers of immune status, were not systematically collected and could therefore not be evaluated as potential confounders. Finally, although we adjusted for the major clinicopathological determinants of both EDIC and outcome, residual confounding inherent to this retrospective cohort study cannot be completely excluded.

## 5 Conclusions

In this retrospective study, EDIC was not only significantly associated with worse BCSS in patients treated with adjuvant RT, but also with worse OS, and more locoregional and distant failure. By integrating multiple OAR parameters into a single immune-relevant metric, EDIC provides complementary information beyond routinely reported OAR doses when evaluating associations between RT and oncologic outcomes. However, the clinical implications of these associations remain hypothesis-generating and require prospective validation.

## CRediT authorship contribution statement

**Stefanie A. de Boer:** Writing – review & editing, Writing – original draft, Visualization, Resources, Project administration, Methodology, Investigation, Formal analysis, Data curation, Conceptualization. **Daan S. Spoor:** Writing – review & editing, Software, Resources, Project administration, Methodology, Investigation, Data curation, Conceptualization. **John H. Maduro:** Writing – review & editing, Resources, Investigation. **Pieter R.A.J. Deseyne:** Writing – review & editing, Resources, Investigation. **Christina T. Muijs:** Writing – review & editing, Resources, Methodology, Investigation, Conceptualization. **Nanna M. Sijtsema:** Writing – review & editing, Resources, Investigation. **Suzanne P.M. de Vette:** Writing – review & editing, Resources, Methodology, Investigation. **Lisanne V. van Dijk:** Writing – review & editing, Software, Resources, Investigation. **Ewoud Schuit:** Writing – review & editing, Visualization, Resources, Investigation, Formal analysis. **Johannes A. Langendijk:** Writing – review & editing, Supervision, Resources, Funding acquisition, Conceptualization. **Anne P.G. Crijns:** Writing – review & editing, Supervision, Resources, Project administration, Methodology, Investigation, Funding acquisition, Data curation, Conceptualization.

## Funding

This study utilizes only data collected at the UMCG as part of the H2020 Euratom research and training program 2014–2018 (Grant agreement No. 755523, MEDIRAD-project, clinicaltrials.gov ID NCT03211442), supplemented with independently obtained additional data from UMCG.

## Declaration of competing interest

No competing financial interests exist for all authors.
