# Supplementary material for: The impact of effective dose to the immune cells on survival in breast cancer patients
Source: Clin Transl Radiat Oncol. 2026 Aug 12;61:101251. doi: 10.1016/j.ctro.2026.101251 (PMC13521130; doi:10.1016/j.ctro.2026.101251)
Supplement: Supplementary file 1 [file mmc1.docx]

**Supplementary material**

**Supplemental Table 1. Correlations EDIC and OARs RT dose**

|  | **MHD** | **MLD** | **ID** |
| --- | --- | --- | --- |
| **EDIC (total population)** | 0.420 | 0.744 | 0.936 |
| ***P*** | <0.001 | <0.001 | <0.001 |
| **N** | 3391 | 3391 | 3391 |
|  | | | |
| **EDIC (left sided BC)** | 0.631 | 0.760 | 0.926 |
| ***P*** | <0.001 | <0.001 | <0.001 |
| **N** | 1764 | 1764 | 1764 |
|  |  |  |  |
| **EDIC (right sided BC)** | 0.473 | 0.798 | 0.948 |
| ***P*** | <0.001 | <0.001 | <0.001 |
| **N** | 1627 | 1627 | 1627 |
| Abbreviations: EDIC, Effective Dose to Immune Cells; MHD, mean heart dose; MLD, mean lung dose; ID, integral dose | | | |

**Supplemental Table 2. Patient Clinical Characteristics at Baseline (n=3423) by EDIC**

| **Characteristic** | | **EDIC 0.0 - 1.5 Gy (n=616)** | **EDIC**  **1.5 - 3.0 Gy (n=2177)** | **EDIC**  **4.0 - 4.5 Gy (n=544)** | **EDIC**  **4.5 -6.2 Gy (n=54)** | ***P-value*** |
| --- | --- | --- | --- | --- | --- | --- |
| Age at breast cancer diagnosis, years | | | | | | |
|  | Categorized, n (%) |  |  |  |  |  |
|  | 18-35 | 17 (2.8) | 36 (1.7) | 17 (3.1) | 4 (7.4) | < 0.001 |
|  | 36-45 | 55 (8.9) | 252 (11.6) | 72 (13.2) | 11 (20.4) |  |
|  | 46-55 | 169 (27.4) | 615 (28.2) | 152 (27.9) | 14 (25.9) |  |
|  | 56-65 | 188 (30.5) | 644 (29.6) | 138 (25.4) | 13 (24.1) |  |
|  | 65-75 | 173 (28.1) | 521 (23.9) | 112 (20.6) | 7 (13.0) |  |
|  | > 75 | 14 (2.3) | 109 (5.0) | 53 (9.7) | 5 (9.3) |  |
| T stage, n (%) | |  |  |  |  |  |
|  | T1 | 500 (81.2) | 1507 (69.2) | 222 (40.8) | 15 (27.8) | < 0.001 |
|  | T2 | 98 (15.9) | 584 (26.8) | 218 (40.1) | 19 (35.2) |  |
|  | T3 | 14 (2.3) | 55 (2.5) | 62 (11.4) | 10 (18.5) |  |
|  | T4 | 4 (0.6) | 31 (1.4) | 42 (7.7) | 10 (18.5) |  |
| N stage, n (%) | |  |  |  |  |  |
|  | N0i+ | 518 (84.1) | 1474 (67.7) | 114 (21.0) | 7 (13.0) | < 0.001 |
|  | N1 | 95 (15.4) | 556 (25.5) | 166 (30.5) | 12 (22.2) |  |
|  | N2 | 2 (0.3) | 100 (4.6) | 163 (30.0) | 16 (29.6) |  |
|  | N3 | 1 (0.2) | 47 (2.2) | 101 (18.6) | 19 (35.2) |  |
| Laterality of the breast, n (%) | |  |  |  |  |  |
|  | Right | 343 (55.7) | 1106 (50.8) | 288 (52.9) | 27 (50.0) | 0.181 |
|  | Left | 273 (44.3) | 1071 (49.2) | 256 (47.1) | 27 (50.0) |  |
| Tumor grade, n (%) | |  |  |  |  |  |
|  | Grade 1 | 203 (33.0) | 516 (23.7) | 72 (13.2) | 2 (3.7) | < 0.001 |
|  | Grade 2 | 290 (47.1) | 996 (45.8) | 251 (46.1) | 24 (44.4) |  |
|  | Grade 3 | 123 (20.0) | 665 (30.5) | 221 (40.6) | 28 (51.9) |  |
| Lympho-vascular space invasion, n (%) | |  |  |  |  |  |
|  | Yes | 62 (10.1) | 309 (14.2) | 201 (36.9) | 23 (42.6) | < 0.001 |
|  | No | 554 (89.9) | 1868 (85.8) | 343 (63.1) | 31 (57.4) |  |
| Subtype, n (%) | |  |  |  |  |  |
|  | HR positive / HER2 negative | 494 (80.2) | 1657 (76.1) | 363 (66.7) | 31 (57.4) | < 0.001 |
|  | HER2 positive | 55 (8.9) | 261 (12.0) | 117 (21.5) | 12 (22.2) |  |
|  | Triple negative | 67 (10.9) | 259 (11.9) | 64 (11.8) | 12 (22.2) |  |
| Chemotherapy, n (%) | |  |  |  |  |  |
|  | Yes | 218 | 993 | 384 | 42 | < 0.001 |
|  | No | 398 | 1184 | 160 | 12 |  |

**Supplemental Figure 1**

**Supplemental Figure 2**

**Figure 2. KM curves for BCSS according to quartiles of OAR dose**

Kaplan–Meier curves for breast cancer–specific survival (BCSS) comparing patients by MHD quartiles (A), MLD quartiles (B) and by ID quartiles (C).
